# Placental mesenchymal dysplasia: A rare case report and literature review

**DOI:** 10.1097/MD.0000000000042663

**Published:** 2025-06-06

**Authors:** Tingting Li, Wei Zhang, Xing Wang, Juntong Li, Hongbing Yang, Huan Yang

**Affiliations:** a Department of Obstetrics, Chongqing University Three Gorges Hospital, Chongqing, China; b Department of General Practice, Shuanghekou Street Community Health Service Center, Wanzhou District, Chongqing, China.

**Keywords:** diagnosis, hydatidiform mole, placental mesenchymal dysplasia, preeclampsia, trophoblastic tumor

## Abstract

**Rationale:**

Placental mesenchymal dysplasia (PMD) is a rare placental disorder that poses diagnostic challenges and is often misdiagnosed as a trophoblastic tumor. While PMD is associated with fetal growth restriction, intrauterine fetal death, and pregnancy-induced hypertension, it does not involve malignant trophoblastic disease. Accurate diagnosis is crucial for optimizing maternal and fetal outcomes.

**Patient concerns:**

A 30-year-old female, gravida 3, para 1, with an uncomplicated medical history, presented with early pregnancy bleeding, which was managed with oral progesterone. Ultrasound at 13 weeks showed normal fetal morphology but a honeycomb-like placental appearance. At 23 weeks, ultrasound revealed fetal growth restriction, thickening of the placenta, and abnormal umbilical artery flow. Magnetic resonance imaging at 32 weeks confirmed abnormal placental masses.

**Diagnoses:**

Ultrasound, magnetic resonance imaging, and genetic testing (noninvasive prenatal testing, amniocentesis, and chromosomal microarray analysis) confirmed normal karyotype and identified distinctive placental abnormalities. Histopathological examination revealed edematous villi, fibromuscular hyperplasia, and amyloid-like protein deposits, consistent with PMD.

**Interventions:**

Prenatal care included enoxaparin sodium and management of fetal distress at 32 weeks with a cesarean section. Magnesium sulfate and dexamethasone were administered for fetal protection.

**Outcomes:**

Both mother and infant were healthy postpartum. The infant had favorable Apgar scores, and the patient’s blood pressure was managed with antihypertensive therapy. Maternal β-human chorionic gonadotropin levels remained normal throughout the pregnancy and postpartum.

**Lessons:**

PMD is an exceptionally rare placental disorder with a low incidence. It often presents with atypical ultrasound findings and can easily be misdiagnosed as a trophoblastic tumor, leading to unnecessary interventions. Diagnosing PMD is challenging, particularly in the absence of specific clinical symptoms. Clinicians must enhance awareness of this rare condition, prioritize early and accurate diagnosis through advanced imaging and histopathology, and differentiate PMD from other placental pathologies to ensure appropriate management and improve maternal-fetal outcomes.

## 1. Introduction

Placental mesenchymal dysplasia (PMD) is a rare placental disorder 1st reported by Moscoso et al in 1991, with an incidence rate of 0.002% to 0.02%.^[1,2]^ Clinical reports indicate a higher incidence in female fetuses.^[3]^ The etiology of PMD is not fully understood, with androgenic/biparental mosaicism being the main hypothesis.^[4,5]^ Clinically, PMD is often associated with fetal growth restriction (FGR),^[6]^ intra uterine fetal distress (IUFD),^[5]^ Beckwith–Wiedemann syndrome (BWS), or preeclampsia,^[7]^ and it increases the risk of amniotic fluid embolism.^[5]^ Most affected fetuses have a diploid karyotype.^[8]^ Due to its ultrasound characteristics of vesicular, enlarged placenta, PMD is often misdiagnosed as a molar pregnancy, making accurate diagnosis challenging. Definitive diagnosis of PMD typically requires a combination of ultrasound imaging, pathological examination, and molecular genetic testing.

In this article, we report a case initially misdiagnosed as a molar pregnancy during gestation and later confirmed as PMD postpartum. We also review the literature on PMD, examining its incidence, clinical features, diagnosis, management, and long-term prognosis.

## 2. Case presentation

**Figure 1.**
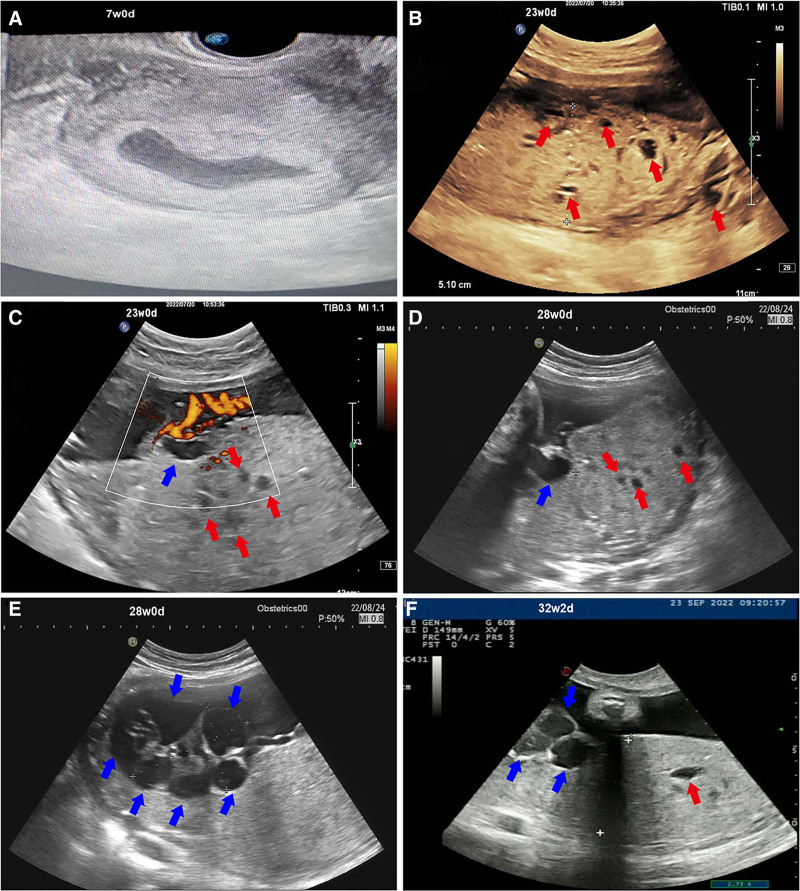
Ultrasound findings of PMD. (A) At 7 wk of gestation, ultrasound reveals an irregularly shaped gestational sac. (B, C) At 23 wk of gestation, ultrasound scans demonstrate placental thickening with extensive diffuse hypoechoic areas, giving a “Swiss-cheese” or “moth-eaten” appearance. Blood flow is observed within the cystic structures, presenting a “ stained glass” sign, and there is dilation of the chorionic plate vessels at the placental-cord interface. (D, E) At 28 wk of gestation, ultrasound scans indicate further thickening of the placenta, with increased dilation of the chorionic plate vessels at the placental-cord interface, resembling a grape-like appearance. (F) At 32 wk and 2 d of gestation, Doppler ultrasound reveals decreased amniotic fluid and abnormal umbilical blood flow. (Red arrows: highlight hypoechoic areas within the placental parenchyma. Blue arrows: vascular tumors at the base of the umbilical cord on the fetal side of the placenta.) PMD = placental mesenchymal mysplasia.

A case of a 30-year-old female, gravida 3, para 1, who conceived spontaneously. The patient had no underlying diseases, took no medications during early pregnancy, and had no history of consanguineous marriage. Prepregnancy screening for toxoplasma, rubella, cytomegalovirus, and herpes simplex virus (TORCH complex) yielded normal results. Twenty days after amenorrhea, the patient tested positive for urinary β-human chorionic gonadotropin (HCG). At 40 days post-amenorrhea, the 1st ultrasound revealed an early intrauterine pregnancy with an irregularly shaped gestational sac (Fig. 1A). Minor vaginal bleeding during 1st trimester pregnancy was managed with oral progesterone for 1 week. Thyroid function tests during 1st trimester pregnancy showed elevated T3 and T4 levels and decreased TSH, which normalized after 1 month without medication.

**Figure 2.**
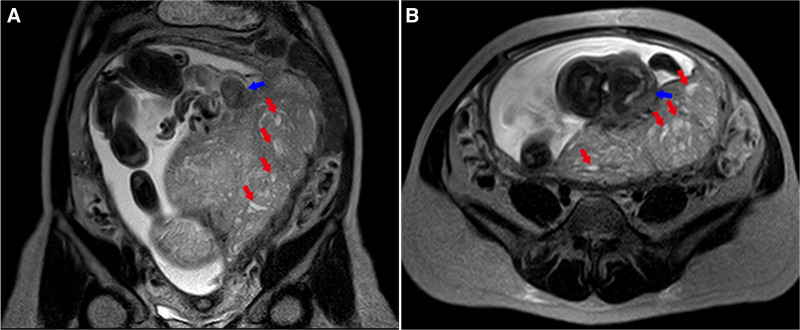
MRI of PMD at 32 wk and 2 d of gestation. (A) T2-weighted sagittal MRI image of PMD. (B) T2-weighted transverse MRI image of PMD. (Red arrows: highlight hypoechoic areas within the placental parenchyma. Blue arrows: vascular tumors at the base of the umbilical cord on the fetal side of the placenta.) MRI = magnetic resonance imaging, PMD = placental mesenchymal mysplasia.

**Figure 3.**
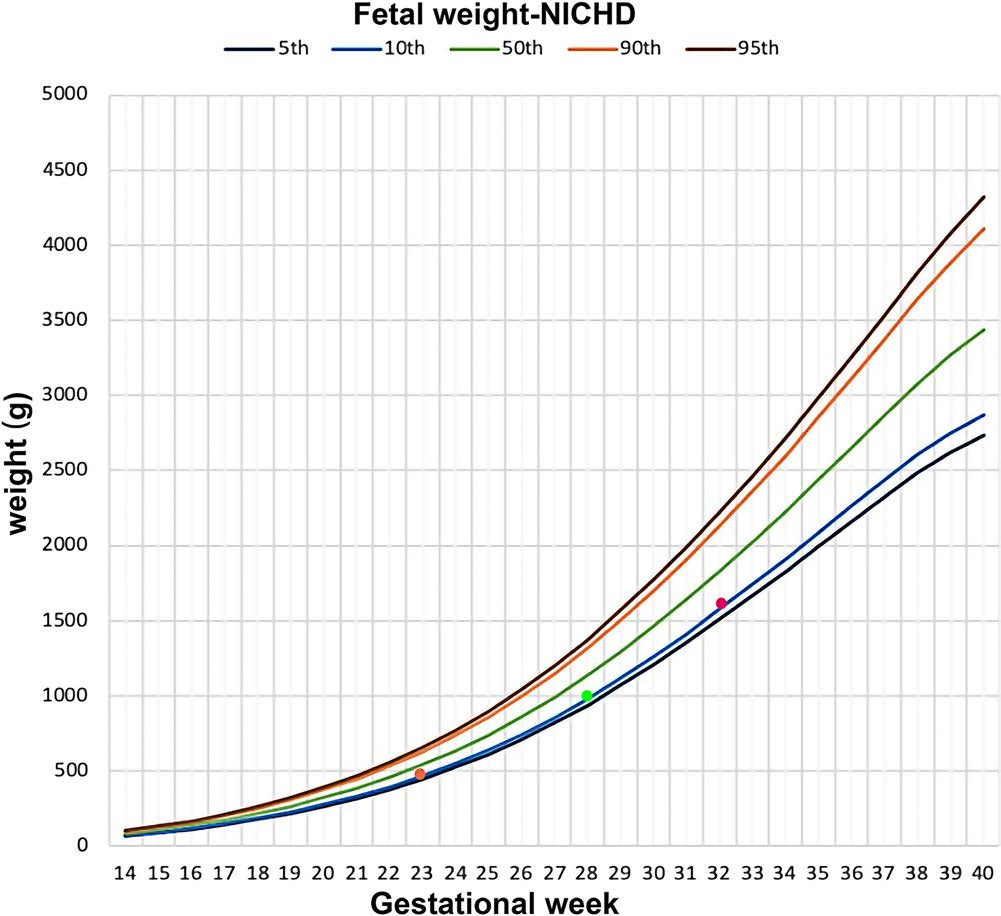
Growth parameter curves at 23 wk, 28 wk, and 32 wk and 2 d.

At 13 weeks of gestation, the patient underwent 1st trimester ultrasound screening at another hospital. The results indicated normal fetal morphology with a nuchal translucency thickness of 1.0 mm, while the placenta exhibited a honeycomb-like appearance. Noninvasive prenatal testing indicated low risk. At 18 weeks ultrasound revealed reduced amniotic fluid volume and a cheese-like change in the placenta. Further amniocentesis confirmed a normal female karyotype, and chromosomal Microarray analysis showed no abnormalities. From 18 weeks of gestation, the patient intermittently received subcutaneous injections of 4000 IU enoxaparin sodium. At 23 weeks, systematic ultrasound revealed increased umbilical artery flow resistance, with fetal biparietal diameter, abdominal circumference, and femur length measurements below gestational age norms. The placenta exhibited thickening with anechoic regions, and the insertion site of the umbilical cord showed cystic anechoic areas, with a “stained glass” blood flow signal within the placental cysts (Fig. 1B,C). The patient opted to continue the pregnancy and received regular outpatient follow-ups. As gestational age advanced, an ultrasound at 28 weeks indicated that the vesicular placental lesions were gradually enlarging, with significant expansion of the chorionic vessels (Fig. 1D,E). At 32 weeks and 2 days, the patient was admitted to the hospital due to concerns of intrauterine fetal distress, and subsequent ultrasound revealed reduced amniotic fluid (Fig. 1F). Further magnetic resonance imaging (MRI) examination revealed multiple abnormal signal masses and nodules within the placenta, with the placenta and the viable fetus located within the same gestational sac (Fig. 2A,B). Throughout the pregnancy, the fetal growth indices were below the 10th percentile (Fig. 3).

**Figure 4.**
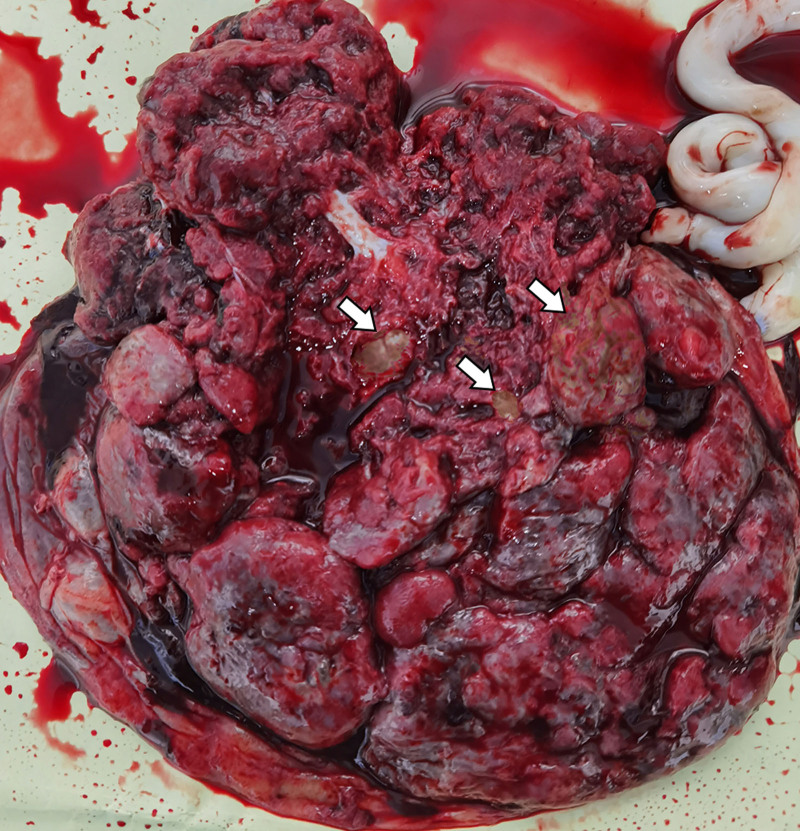
Macroscopic findings of the placenta. Multiple variably sized pale-yellow vesicles within the placental parenchyma (white arrows).

During hospitalization, the patient received intravenous magnesium sulfate to protect the fetal nervous system. Simultaneously, a course of dexamethasone was administered via intramuscular injection to promote fetal lung maturation. Subsequently, a cesarean section was performed to terminate the pregnancy. Intraoperatively, it was found that the amniotic fluid was contaminated with grade III. A female infant weighing 1530 g was delivered, with Apgar scores of 8-9-9 at 1 minute, 5 minutes, and 10 minutes, respectively. Examination of the placenta revealed dimensions of approximately 29 × 26 × 6 cm, with a weight of approximately 680 g and a thickness of 6 cm. Multiple variable-sized pale-yellow vesicular structures were visible on the maternal surface of the placenta. The umbilical cord insertion was central, and the placental maternal surface near the cord base showed protruding grape-like vesicular structures (Fig. 4). Postpartum, the patient experienced elevated blood pressure, peaking at 160/120 mm Hg, and received treatment including antihypertensive and antispasmodic therapies.

**Figure 5.**
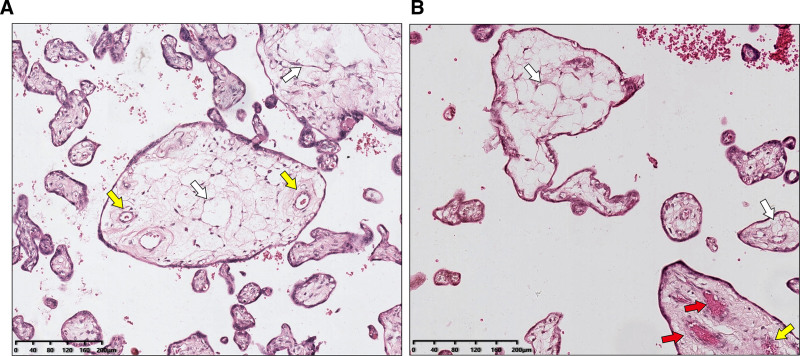
H&E staining of PMD. (A) Histopathological analysis indicates enlarged chorionic villi with edematous expansion and cistern-like structures (white arrows). Some chorionic villous vessels exhibit significant fibromuscular hyperplasia and thickened walls (yellow arrows). (B) Local occlusion of vascular lumens is observed, surrounded by poorly developed vessels with thick walls and amyloid protein deposition around the villi (red arrows). There is no evidence of typical trophoblastic proliferation or inclusion bodies within interstitial cells. Scale bars: 200 µm. H&E = hematoxylin-eosin staining, PMD = placental mesenchymal mysplasia.

**Figure 6.**
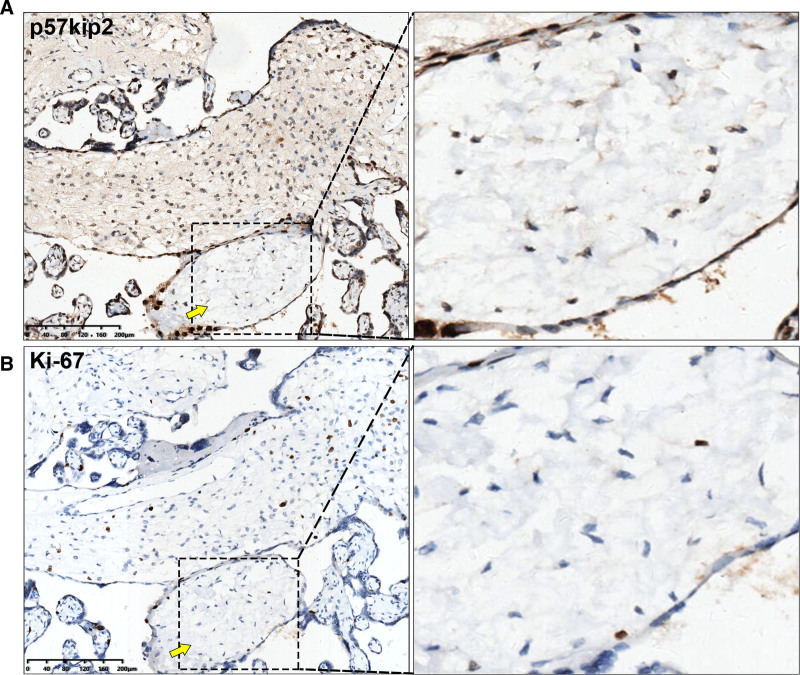
IHC staining of PMD. (A) P57kip2 immunostaining exhibits minimal expression in edematous chorionic villi (yellow arrow), contrasting with positive staining observed in terminal villous tissues. (B) Ki-67 immunostaining shows reduced expression in poorly developed interstitial edematous tissue and normal chorionic villous tissue. Scale bars: 200 µm. PMD = placental mesenchymal mysplasia.

Histopathological examination revealed edematous stem villi, characterized by some villous vessels exhibiting marked fibromuscular hyperplasia and localized luminal occlusion (Fig. 5A). Surrounding these findings were poorly developed vessels with thickened walls and deposits of amyloid-like protein around the periphery of the villi. There was no evidence of typical syncytiotrophoblast proliferation or stromal cell inclusions (Fig. 5B). Immunohistochemical analysis demonstrated minimal expression of anti-*p57kip2* staining in the edematous stroma and stem villi, contrasting with positive staining in normal villi and terminal villous tissue (Fig. 6A). Anti-*Ki-67* staining revealed low expression in both the poorly developed stroma of edematous tissue and normal villous tissue (Fig. 6B).

**Figure 7.**
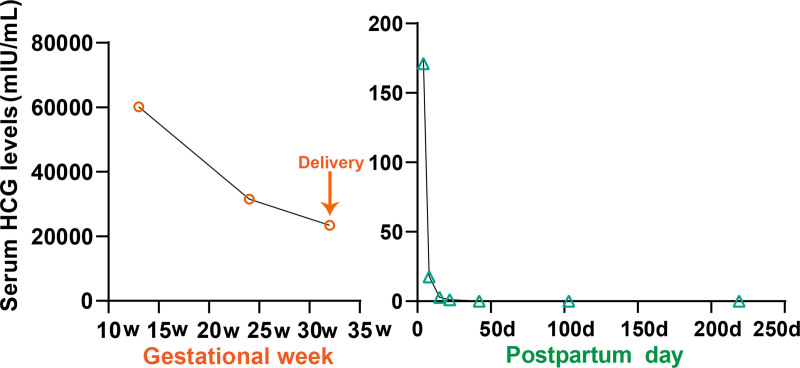
Serum β-HCG levels during pregnancy and postpartum follow-up. HCG = human chorionic gonadotropin.

The premature newborn had a favorable prognosis, showing no visceral or biochemical abnormalities such as thrombocytopenia or anemia. Throughout the pregnancy, maternal β-HCG levels remained within normal ranges and turned negative 2 weeks postpartum (Fig. 7). At the 1-year postpartum follow-up, both mother and infant remained healthy without any complications. Publication of this case report was authorized with informed consent from the patient.

## 3. Discussion

PMD is a rare and complex placental disorder initially documented by Moscoso et al in 1991.^[1]^ Key features of PMD include placental enlargement, vesicular formations, and dilation of chorionic vessels.^[9]^ These characteristics often lead to misdiagnosis during prenatal assessments, with PMD frequently confused with partial hydatidiform mole (PHM) or complete hydatidiform mole coexisting with a normal fetus (CHMF).^[10,11]^ Differential diagnosis is also necessary to distinguish PMD from spontaneous abortion accompanied by placental edema and placental chorioangioma (Table 1). Importantly, PMD does not carry the risk of malignant trophoblastic disease and allows for the birth of a healthy fetus. Therefore, accurate differentiation between PMD and hydatidiform mole is critical due to significant differences in their management and prognosis.^[12]^

**Table 1:** Key points for the diagnosis and differential diagnosis of PMD.

Diagnostic item	PMD	PHM	CHMF
Imaging			
Ultrasound	Placental thickening with various-sized liquid cysts interspersed with normal placental tissue (appearing “cheese-like” or “moth-eaten”)	Placental thickening with uniformly sized liquid cysts (appearing “grape-like”)	Placental thickening with uniformly sized liquid cysts (appearing “grape-like”)
Doppler	Blood flow signals visible within cystic (appearing “stained glass” appearance)	No blood flow within cystic	No blood flow within cystic
MRI	A normally developing fetus often shares the same gestational sac as the diseased placenta	Occasionally, a developing fetus is visible	A normally developing fetus is often in a separate gestational sac from the diseased placenta; bleeding may be present within the cysts
Serum markers	Normal or slightly elevated HCG; elevated MSAFP	Elevated HCG	Elevated HCG
Karyotype	Amniocentesis shows diploid; chorionic villus sampling shows androgenetic/biparental mosaicism	Most cases are diploid	Triploid
Pathology			
Gross morphology	Enlarged placenta, total weight > 95%; cystic, bubbly structures; diffuse vascular proliferation resembling hemangioma-like changes	Cystic, bubbly placenta	Cystic, bubbly placenta
Microscopic	Hydropic swelling of the villous stroma, normal terminal villi; thickened vessel walls with thrombosis; lack of trophoblast proliferation	Hydropic swelling of terminal villi; trophoblast proliferation	Hydropic swelling of terminal villi; trophoblast proliferation
Immunohistochemistry	P57 kip2 negative staining in hydropic stroma, positive staining in terminal villi; Ki-67 negative staining	P57 kip2 positive staining; Ki-67 positive staining	P57 kip2 negative staining; Ki-67 positive staining

CHMF = complete hydatidiform mole coexisting with a normal fetus, HCG = human chorionic gonadotropin, MRI = magnetic resonance imaging, MSAFP = maternal serum alpha-fetoprotein, PHM = partial hydatidiform mole, PMD = placental mesenchymal mysplasia.

### 3.1. Incidence

The precise incidence rate of PMD remains unclear, with only slightly over 100 cases documented in the literature to date. According to historical reports, its incidence rate is estimated to be approximately from 0.002% to 0.02%,^[2]^ with a male-to-female ratio reported between 1:3.6 and 4.0.^[3]^ In contrast, the incidence of hydatidiform mole is estimated between 0.1% and 0.3%.^[13]^ Underreporting of PMD stems from its frequent misclassification with other placental disorders and the limited number of placentas subjected to comprehensive pathological and genetic examination.^[3]^ Additionally, inadequate clinical awareness of PMD contributes to both misdiagnosis and potential underestimation of its true incidence rate.

### 3.2. Etiology

The precise etiology of PMD remains unclear, yet several predominant hypotheses have been proposed. Firstly, congenital malformations of the mesenchyme are considered a potential cause.^[14]^ Gerli et al suggested that PMD originates from mesodermal dysplasia, as mesenchymal cells found in villi, chorionic tumors, hemangiomas, and chorionic blood vessels also are found in normal chorionic mesoderm.^[15]^ Similar to other placental proliferative disorders, acidic mucopolysaccharides have been identified in the chorionic stem cells of PMD placental villi.^[16]^

Secondly, molecular disruptions affecting the 11p15.5 chromosomal imprinting genes associated with BWS also play a significant role.^[17]^ Approximately 25% of PMD cases reportedly co-occur with BWS.^[18]^ Features of BWS include loss of methylation of *DMR2*, hypermethylation of *DMR1* on the maternal allele, mutations in the maternal *CDKN1C* gene, and biallelic expression of normally paternally imprinted genes. Furthermore, dysregulation of *IGF2* – typically paternally expressed – or mosaic paternal uniparental disomy of 11p15.5 may also play a role.^[19–22]^

Lastly, among reported PMD cases, androgenetic/biparental mosaicism represents the most common etiology, characterized as an atypical non-diploid abnormality.^[4,5]^ Errors during the 1st cleavage of the zygote lead to the production of a normal cell containing both maternal and paternal genes and a non-diploid cell containing only paternal genes. During subsequent cell divisions, androgenetic cells localize to the chorionic membrane and placental vessels, interspersed with normal cells.^[12,23]^ These cells primarily reside in the mesenchyme of chorionic villi, membranes, and vessels, but are absent in the trophoblast layer. Conversely, complete hydatidiform moles lack maternal DNA in trophoblast cells, whereas PHM contain 2 paternal chromosome sets, leading to trophoblast hyperproliferation.^[12]^

### 3.3. Diagnosis and differential diagnosis

When an enlarged cystic placenta is identified during pregnancy, consideration should be given to the possibility of PMD. It is crucial to conduct a comprehensive assessment from various perspectives to distinguish between PHM, CHMF, spontaneous abortion associated with placental edema changes, placental chimerism, and placental chorioangioma. These conditions present significantly varied prognoses for both the fetus and the pregnant woman.^[24]^

#### 3.3.1. Ultrasound

Vaisbuch et al recommended detailed “dissection” ultrasound examination of the placenta upon detecting placental enlargement.^[25]^ Typical ultrasound features of PMD include a cystic placenta with hypoechoic areas, alongside placental enlargement or thickening. Ohira et al reported that approximately 70% of cases are diagnosed as cystic placenta between 13 and 20 weeks of gestation.^[2]^ As pregnancy progresses, cysts gradually increase, manifesting as multiple hypoechoic spaces resembling a “Swiss cheese” or “moth-eaten” appearance.^[26]^ By 3rd trimester pregnancy, around 90% of placentas exhibit villous mesenchymal vascular dilation.^[14]^ Two-dimensional ultrasound struggles to differentiate PMD, PHM, and CHMF due to their similar appearances as thickened placentas with widespread vesicular structures on imaging.^[27]^ Studies indicate that 3-dimensional ultrasound offers clearer delineation of these conditions. It has been observed that cysts in PHM are uniformly round in size and shape, whereas those in PMD vary in size and shape, representing a distinctive ultrasound feature.^[27]^ In contrast, distinguishing PMD from CHMF is relatively straightforward, as placental lesions in PMD occur within the same gestational sac as the fetus, whereas CHMF involves a separate sac for the diseased placenta and fetus.^[12,27]^

Furthermore，different subtypes of gestational trophoblastic disease exhibit distinct sonographic characteristics. Invasive mole and choriocarcinoma typically present as heterogeneous masses within the myometrium, with mixed echogenicity, multiple internal cystic spaces, and prominent vascularity on Doppler imaging. These lesions often lead to myometrial thickening, contour irregularity, and in some cases, arteriovenous fistula formation. In contrast, placental site trophoblastic tumor and epithelioid trophoblastic tumor appear as well-defined intramyometrial nodules containing both cystic and solid components. Epithelioid trophoblastic tumor is often characterized by a sharply demarcated hypoechoic halo and marked peripheral Doppler signals, which assist in distinguishing it from other trophoblastic lesions.^[28]^

#### 3.3.2. Doppler

Doppler ultrasound can be utilized as an auxiliary method for assessing blood flow in the cystic areas of the placenta.^[12]^ In late pregnancy, employing a low pulse repetition frequency setting to observe low speed blood flow can effectively differentiate between molar pregnancy and PMD.^[29]^ In PMD, there is extensive arterial or venous blood flow turbulence within the placental lesions, primarily located beneath the chorionic plate. This phenomenon is often described as having a “stained glass” appearance and is attributed to the gradual dilation of chorionic arteries and veins, forming aneurysms.^[30]^ Conversely, the placental cysts in molar pregnancy are due to edema and, therefore, do not exhibit blood flow signals.^[27]^ Moreover, color Doppler is beneficial in distinguishing PMD from other placental abnormalities with similar ultrasound vesicular features, such as chorioangiomas (characterized by large/increased blood vessels), hydropic spontaneous abortion (avascular), and subchorionic hematoma (avascular).^[31,32]^ Although PMD placentas generally show increased blood flow, the level of blood flow can vary among patients. In molar pregnancy, while the edema itself may not show blood flow, abnormal intervillous circulation may still be observed. In cases complicated by intervillous hemorrhage, color Doppler may also reveal blood flow in the edematous areas.^[33]^ Therefore, a combination of color Doppler and MRI is recommended to help differentiate between these various conditions.

#### 3.3.3. Magnetic resonance imaging

MRI offers distinct advantages in differentiating PMD from molar pregnancy, including its ability to obtain high-quality images regardless of maternal body habitus and even in cases of oligohydramnios.^[34]^ MRI can provide comprehensive visualization of the placenta, clearly delineating the location and extent of cystic lesions.^[2]^ In PMD, lesions are located within the gestational sac and are associated with the developing fetus, whereas in CHMF, lesions are situated outside the gestational sac.^[35]^ Additionally, MRI can identify cystic hemorrhage, a common feature in molar pregnancy but rare in PMD.^[35]^ When ultrasound and Doppler are insufficient for determining the position and extent of cysts, MRI can be particularly beneficial.

#### 3.3.4. Serum markers

To some extent, imaging characteristics alone are insufficient to completely differentiate PMD from other placental multicystic diseases. In such instances, serum biomarkers provide significant diagnostic guidance. PMD typically presents with normal or mildly elevated maternal serum β-HCG levels and high levels of maternal serum alpha-fetoprotein (MSAFP).^[14,36]^ In contrast, molar pregnancies are characterized by markedly elevated β-HCG levels and normal MSAFP levels.^[12]^ The elevated MSAFP in PMD cases is hypothesized to result from the increased placental surface area, vascularization, and volume, leading to enhanced transfer of AFP into the maternal circulation.^[14,37]^ Conversely, the excessive proliferation of trophoblastic cells in molar pregnancies causes the abnormal elevation of β-HCG levels.^[3,23]^

#### 3.3.5. Karyotype

When ultrasound examination reveals placental enlargement, fetal karyotype analysis becomes a crucial step in differentiating PMD from molar pregnancy.^[2,3]^ Most pregnancies with PMD involve female fetuses with a normal diploid karyotype (46XX).^[8]^ The *VEGF-D* gene, located on the X chromosome (Xp22.31), is associated with angiogenesis,^[38]^ and mutations at this locus are often found in pregnant women with PMD. This suggests a possible reason why PMD predominantly occurs in female fetuses.^[19,38]^ PHM are typically associated with triploid fetuses, resulting from 2 sets of paternal chromosomes.^[39]^

Given that complete molar pregnancies, CHMF, and hydropic spontaneous abortions are usually diploid, the presence of an abnormal karyotype necessitates considering the possibility of a PHM.^[40]^ However, fetal chromosomal karyotype analysis alone is insufficient to distinguish PMD from molar pregnancy, as androgenetic/biparental mosaicism plays a significant role in PMD. Arigita et al found that while the fetal karyotype in PMD is usually normal (diploid), the PMD placenta often exhibits mosaicism, with mesodermal and vascular tissues of the chorion being biparental diploid (containing double paternal chromosomes). These diploid cells are interspersed with normal cells, forming a mosaic pattern within the placenta. Therefore, Arigita et al recommend that if PMD is suspected, prenatal diagnosis through chorionic villus sampling or placental biopsy should be performed.^[41]^

#### 3.3.6. Pathology

Diagnosing PMD involves thorough macroscopic and microscopic pathological examination of the placenta postpartum. Macroscopically, PMD typically presents with placental enlargement exceeding the 95th percentile in weight.^[42]^ The chorionic villi exhibit diffuse cystic vesicles and aneurysmal vessels scattered throughout the normal parenchyma.^[20,43]^ Additionally, PMD often accompanies umbilical cord anomalies such as excessive length, single umbilical artery, excessive twisting, coiling, and eccentric insertion.^[14]^ Microscopically, PMD is characterized by expanded chorionic villi stalks displaying a mucinous or edematous appearance, while terminal villi maintain a normal morphology.^[20]^ Pathological features include a lack of trophoblastic cell layer proliferation, presence of central cisterns, thickening of fibromuscular chorionic villi vessel walls, and intravascular thrombosis.^[44]^ In contrast, while molar pregnancies also exhibit numerous cystic vesicles in the placenta, distinguishing features include the presence of trophoblastic cell layer proliferation and edema in terminal villi.^[3,8]^

For suspected PMD cases, immunohistochemical (IHC) staining of the placenta is essential. *p57kip2*, a maternally imprinted gene,^[39]^ plays a critical role in differentiating PMD. Due to the androgenetic/biparental mosaicism characteristic of PMD, edematous chorionic villi stalks lack maternal genomic imprinting. Therefore, IHC staining for *p57kip2* typically reveals positive staining in terminal villi and negative staining in edematous chorionic villi stalks, as confirmed in our cases. Conversely, PHM with maternal genomic imprinting show positive *p57kip2* staining, while complete molar pregnancies with only paternal genomic imprinting show negative *p57kip2* staining.^[39]^

Both normal and dysmorphic villi typically exhibit positivity for cytokeratin and vimentin; however, smooth muscle actin is expressed in normal villi but is absent in dysmorphic villi. Consistent with our findings, low proliferative rates are indicated by negative *Ki-67* IHC staining.^[22,24]^ Furthermore, Takahashi et al reported strong staining of β*-catenin*, *Wnt3a*, and *Wnt5a* in trophoblast cells of PMD, associating with cellular proliferation and differentiation. Additionally, increased expression of *VEGF-D* and *DKK-1* was observed, implicating their involvement in angiogenesis.^[4]^ These genetic markers may provide insights into underlying mechanisms of PMD occurrence, although specific causal factors require further validation.

### 3.4. Implications

#### 3.4.1. Fetal implications

PMD is associated with a variety of fetal diseases and syndromes. Reported conditions and complications include hematologic disorders (such as anemia and thrombocytopenia), BWS, liver tumors, FGR, preterm birth, and IUFD.^[20,45]^ Fetal anemia and thrombocytopenia often manifest when abnormalities in fetal middle cerebral artery Doppler suggest possible anemia during pregnancy.^[20,45]^ However, the mechanisms underlying fetal anemia and thrombocytopenia in PMD remain unclear. Some studies suggest that these complications may result from abnormal placental blood flow distribution and vascular dilatation, leading to secondary microangiopathic hemolytic phenomena.^[22,46,47]^

PMD is associated with BWS in 25% of cases.^[7,18,22]^ BWS is a genetic disorder characterized by abnormal expression of imprinted genes on chromosome 11p15.5, presenting with features such as macrosomia, visceral organomegaly, hemihypertrophy, macroglossia, omphalocele, insulin resistance, and adrenal cytomegaly, detectable either prenatally or in infancy.^[32,48]^ Given that the 11p15.5 locus includes genes like *IGF2*, *CDKN1C,* and *DMR*, some researchers speculate that abnormal genetic imprinting at this locus may predispose to PMD in BWS.^[7]^

Liver tumors, particularly mesenchymal hamartoma of the liver (MHL), are also a complication of PMD but are infrequent. Since the 1st reported case in 1983, there have been 19 documented cases of PMD possibly complicating MHL.^[7]^ Although MHLs are benign liver tumors, they may grow rapidly, potentially leading to fetal cardiovascular failure and poor prognosis.^[21,49]^ Therefore, thorough placental ultrasound examination is essential for obstetricians when fetal liver tumors are detected, to determine whether PMD is concurrently present.

The most common fetal complications associated with PMD include FGR, preterm birth, and IUFD, with incidence rates ranging from 33% to 72%, 53% to 64%, and 13% to 18%, respectively.^[3,26,40]^ Pham et al propose that diffuse vascular anomalies can lead to varying degrees of shunting and obstructive thrombosis, resulting in compromised fetal perfusion and chronic hypoxia.^[19]^ Additionally, increased expression of the *VEGF-D* gene on chromosome Xp22.31 in PMD placenta is linked to common vascular and follicular vascular proliferation, potentially contributing to chronic fetal hypoxia.^[19,38]^ Rupture of improperly dilated chorionic villous vessels or significant thrombotic events within the umbilical cord may also precipitate sudden IUFD.^[50]^ Complications affecting both fetus and mother often necessitate preterm delivery in the majority of PMD cases, either due to medical indications or spontaneous preterm labor.^[40]^

#### 3.4.2. Maternal implications

The maternal implications of PMD remain poorly understood due to limited available literature. However, Kodera et al reported that approximately 12.8% of PMD cases are associated with maternal hypertensive disorders, including gestational hypertension, preeclampsia, eclampsia, and HELLP syndrome.^[3,26]^ The pathogenesis of these conditions often involves inadequate invasion of trophoblasts and insufficient remodeling of spiral arteries in the placenta, leading to placental ischemia and hypoxia. These mechanisms bear similarity to those observed in PMD, suggesting a need for monitoring and preventive measures against conditions such as preeclampsia in PMD patients.^[51]^ Additionally, the increased placental size in PMD has occasionally been linked to complications such as placenta previa, postpartum hemorrhage, and amniotic fluid embolism.^[5,52]^

## 4. Management

Due to the rarity of PMD, standardized expert opinions on its management are currently lacking (Table 2). Timely and accurate identification of PMD is crucial, as clinicians often lean towards terminating pregnancy when encountering an enlarged cystic placenta, fearing complications from malignant trophoblastic tumors. This inclination may contribute to the limited documentation of cases to date. However, comprehensive prenatal care typically yields favorable outcomes in PMD cases, making termination of pregnancy unwise.^[3]^

**Table 2:** Common clinical manifestations and management points of PMD.

Management points	Manifestations	
	Fetal	Maternal
Ultrasound and Doppler	FGR, BWS, IUFD	Placenta previa, polyhydramnios
Middle cerebral artery Doppler	Fetal anemia, intrauterine fetal distress	–
Non-stress test of electronic fetal monitoring	Intrauterine fetal distress, preterm labor	–
Amniocentesis	Fetal chromosomal abnormalities	–
Blood pressure and urine protein	–	Preeclampsia, eclampsia
HCG	–	Malignant trophoblastic tumors

BWS = Beckwith–Wiedemann syndrome, FGR = fetal growth restriction, HCG = human chorionic gonadotropin, IUFD = intra uterine fetal distress, PMD = placental mesenchymal dysplasia.

When doctors encounter suspicious ultrasound findings, it is essential to differentiate PMD from PHM or CHMF, given the distinct treatments, outcomes, complications, and postpartum monitoring requirements. Initially, this differentiation can be achieved through prenatal imaging and biochemical markers. Typical ultrasound and Doppler characteristics of PMD include an enlarged cystic placenta with a coexisting viable fetus within the same gestational sac. These features are often characterized by rich vesicular blood flow and a “stained glass” appearance.^[53]^ Consideration of MRI for enhanced imaging to localize the lesion may be necessary.^[34]^ Additionally, normal β-HCG levels and elevated MSAFP levels associated with PMD can facilitate diagnosis.^[53]^ Furthermore, invasive testing can provide valuable information on fetal karyotype, which typically appears normal in most PMD cases. In instances where triploidy is detected, consideration of PHM is warranted, with potential implications for continuing the pregnancy, thus suggesting termination.^[39]^

In evaluating PMD, healthcare providers must ensure clear communication regarding associated risks, including FGR, intrauterine fetal death, and pregnancy-induced hypertension.^[3]^ Enhanced monitoring during pregnancy should encompass late-term serial growth scans and fetal health assessments, including middle cerebral artery Doppler evaluation for fetal anemia.^[3,34,50]^ Vigilant monitoring of prodromal signs of preeclampsia, such as blood pressure, urine protein, complete blood count, liver function, and renal function, is essential. In certain scenarios, cesarean section may be the preferred delivery mode. Furthermore, following delivery of suspected PMD cases, placental tissue should undergo histopathological analysis to confirm diagnosis, with typical pathological features including a lack of proliferative trophoblastic layers. Despite reports largely indicating no association between PMD and malignant trophoblastic tumors, there have been isolated cases of choriocarcinoma diagnosed months postpartum.^[54]^ Consequently, follow-up protocols for PMD patients should encompass close monitoring of postpartum HCG levels.

## 5. Conclusion

**Figure 8.**
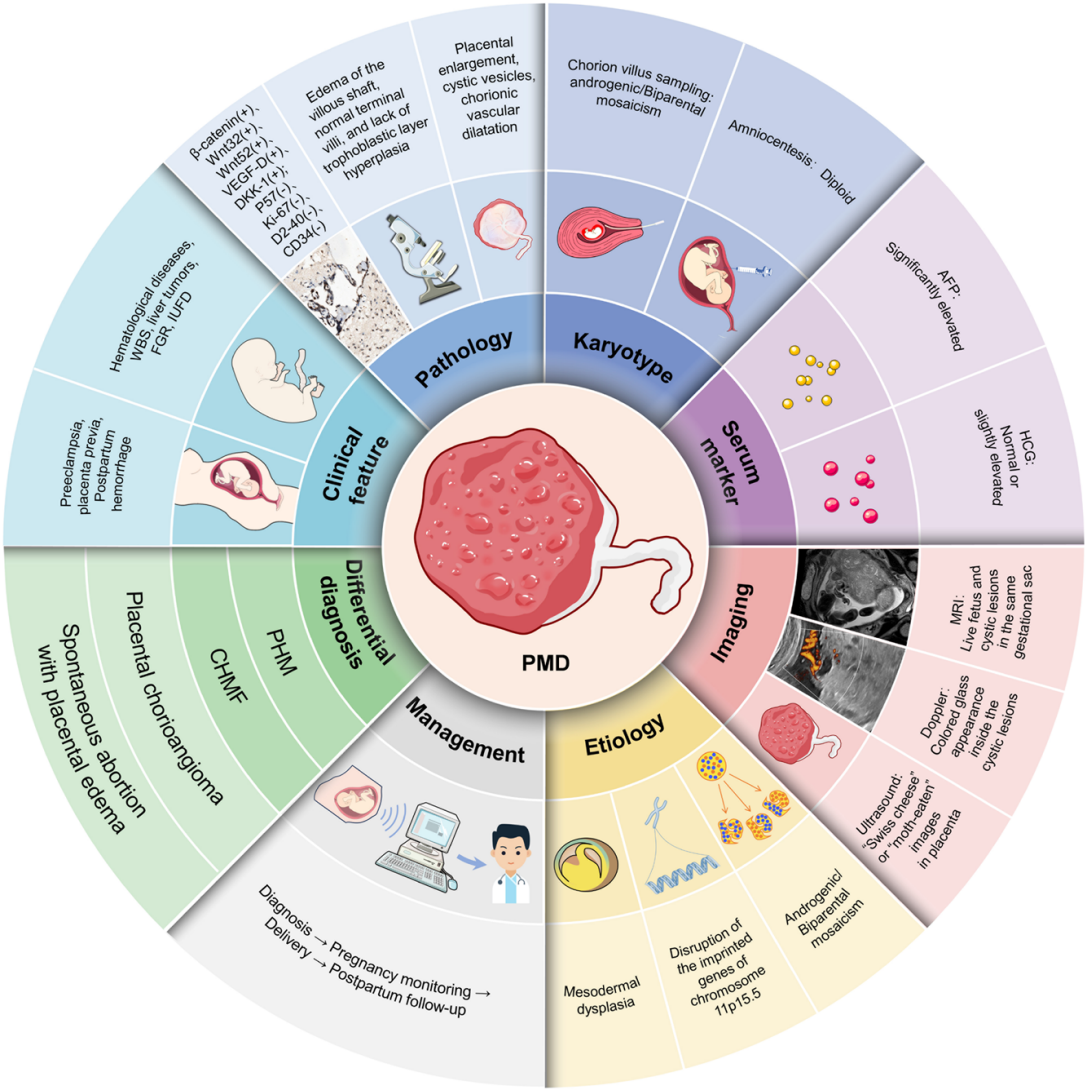
Full landscape of diagnosing and managing PMD. PMD = placental mesenchymal mysplasia.

In conclusion, PMD is a rare condition that is frequently misdiagnosed as a molar pregnancy due to limited awareness, often resulting in unnecessary pregnancy termination. Unlike molar pregnancies, PMD does not carry the risk of malignant trophoblastic disease, leading to significantly different maternal and fetal outcomes. Therefore, when prenatal imaging reveals cystic placental changes, clinicians should include PMD in the differential diagnosis and pursue further characterization with MRI to delineate lesion morphology and vascular flow patterns. Concurrent assessment of serum β-HCG concentrations and fetal karyotype facilitates exclusion of gestational trophoblastic disease and chromosomal anomalies. Definitive diagnosis ultimately relies on histopathological examination of villous tissue. By thoroughly counseling patients regarding PMD’s generally favorable prognosis and absence of malignant potential, clinicians can avoid inadvertent termination of potentially viable pregnancies (Fig. 8).

## Acknowledgments

We would like to thank all the nurses and doctors and other staff in the obstetrics department of Chongqing University Three Gorges Hospital who provided excellent care and accurate data recording to the patients.

## Author contributions

**Conceptualization:** Huan Yang.

**Data curation:** Tingting Li.

**Formal analysis:** Tingting Li, Wei Zhang.

**Funding acquisition:** Huan Yang.

**Investigation:** Wei Zhang, Juntong Li.

**Methodology:** Xing Wang.

**Project administration:** Hongbing Yang.

**Software:** Xing Wang.

**Validation:** Juntong Li.

**Writing – original draft:** Hongbing Yang.

**Writing – review & editing:** Huan Yang.
